# A Review on Relationship Between Charcot Neuroarthropathy and Diabetic Patients

**DOI:** 10.7759/cureus.50988

**Published:** 2023-12-23

**Authors:** Himani Bansod, Anil Wanjari, Omkar Dumbhare

**Affiliations:** 1 Medicine, Jawaharlal Nehru Medical College, Datta Meghe Institute of Higher Education and Research, Wardha, IND; 2 Cardiology, Jawaharlal Nehru Medical College, Datta Meghe Institute of Higher Education and Research, Wardha, IND; 3 Genetics, Jawaharlal Nehru Medical College, Datta Meghe Institute of Higher Education and Research, Wardha, IND

**Keywords:** diabetes-related foot disorders, musculoskeletal complications, peripheral neuropathy, diabetic patients, charcot neuroarthropathy

## Abstract

Charcot Neuroarthropathy (CN) is a complex and incapacitating disorder characterized by neuropathy, progressive deformity, and joint destruction. It is of substantial interest within the diabetic population as this ailment chiefly affects individuals with diabetes. The pathophysiology of CN is multidimensional, connecting peripheral neuropathy, repetitive trauma, and autonomic dysfunction. The review analyses the mechanisms directing the development of CN, emphasizing the influence of diabetes in individuals who lean toward this condition. Clinical presentation and diagnosis of CN in diabetic patients present unique challenges. Complex clinical features have also been discussed, including joint deformities, insidious onset, and painless swelling, which mimic other musculoskeletal conditions. The diagnostic approaches, involving clinical examination and radiological imaging, are analyzed for early and accurate diagnosis. Risk factors and epidemiology emphasize the prevalence of CN within the diabetic population and draw attention to common risk factors contributing to its development. Significant factors such as glycemic control, duration of the disease, and type of diabetes are important in estimating an individual's risk for CN. Complications, such as foot ulcers and amputations, provide an understanding of the severe outcome of this condition on patients' quality of life. Management approaches and treatment involving conservative and surgical approaches are reviewed in depth. A multidisciplinary approach to patient care is emphasized, given the complex nature of CN and the comorbidities existing in diabetic individuals. Prognosis and prevention comprise approaches for mitigating the risk of CN in diabetic patients, such as glycemic control, regular foot examinations, and patient education. This thorough review aims to outline the intricate relationship between CN and diabetes, offering an understanding of pathophysiology, clinical complexities, diagnostic nuances, treatment modalities, and prevention strategies.

## Introduction and background

Charcot Neuroarthropathy (CN), also known as neuropathic osteoarthropathy, charcot foot, or simply charcot joint, is a rare, weakening condition primarily affecting individuals with diabetes mellitus. This disorder is characterized by progressive neuropathy, deformity, and joint destruction, leading to substantial morbidity and functional impairment [1]. The term "Charcot" is derived from the French neurologist Jean-Martin Charcot [2]. In diabetes, CN signifies a patent clinical presentation indicated by impaired proprioception, autonomic dysfunction, and profound sensory neuropathy. It classically manifests in the lower extremities, particularly the foot and ankle, but other joints are also involved [3]. Diabetes mellitus, an omnipresent metabolic disorder associated with various complications that impact nearly every organ system, affects millions of individuals worldwide [4]. One obscure yet intensely incapacitating complication is CN, which impacts most of the diabetic population. This condition creates a significant healthcare challenge and often remains underdiagnosed. Understanding the connection between CN and diabetes is paramount for several reasons. Firstly, for making early diagnosis and intervention, vital to preventing disease development, the disorder's clinical presentation often mimics other musculoskeletal disorders such as rheumatoid arthritis and gout [5]. The painless nature and insidious onset of CN produces diagnostic challenges, leading to delayed diagnosis in many cases. CN results in severe joint instability, deformities, foot ulcers, and lower extremity amputations if left untreated. The long-term outcomes of such complications are physically incapacitating and psychologically and emotionally disturbing for affected individuals [1,3]. The costs accompanying prolonged hospitalizations, rehabilitation, and treatment of complications are significant. By understanding the relationship between CN and diabetes and implementing preventive measures, the healthcare burden can be significantly reduced.

## Review

Methodology

This systematic review strictly followed the guidelines outlined in the Preferred Reporting Items for Systematic Reviews and Meta-Analyses (PRISMA). Data collection occurred in August 2023 by searching various electronic databases, including PubMed, Google Scholar, ScienceDirect, PubMed Central, and Cochrane Data. These searches involved the utilization of keywords like "Charcot Neuroarthropathy," "Diabetic patients," "Neuropathy," "Diabetes-related foot disorders," and "Pathophysiology," both independently and in various combinations. A total of 2324 cases were identified through these keyword-based searches. Subsequently, the articles were assessed based on their titles, abstracts, and their relevance to the research inquiry. Inclusion and exclusion criteria were applied to refine the selection further to identify articles pertinent to the study. The databases were most recently accessed in August 2023.

Search Sources/Search Strategy

In PubMed, Google Scholar, ScienceDirect, PubMed Central, and Cochrane data, we used the MeSH strategy we obtained:("Charcot Neuroarthropathy/pathophysiology" [Majr] OR "Charcot Neuroarthropathy/clinical features" [Majr] OR "Charcot Neuroarthropathy/sensory neuropathy" [Majr] OR "Charcot Neuroarthropathy/Autonomic nervous system dysregulation" [Majr] OR "Diabetes/immune dysregulation" [Majr] OR "Charcot Neuroarthropathy/Diabetes" [Majr] OR "Charcot Neuroarthropathy/prevention" [Majr]); for the treatment it was: ("Treatment/conservative" [Majr] OR "Treatment/surgical" [Majr] OR "Intervention strategies/Lifestyle modification" [Majr] OR "Conservative treatment/offloading" [Majr]). We acquired the most relevant research papers and employed various combinations using Boolean operators such as "AND" and "OR".

Inclusion and Exclusion Criteria

Our selection criteria primarily encompassed English-language papers published in the past three decades that directly pertained to the core inquiries of this review article. We specifically sought review studies, randomized clinical trials, and observational studies. Conversely, papers written in languages other than English, those unrelated to the research questions, and those addressing subjects beyond the scope of CN and its intricate association with diabetes were deliberately excluded from consideration.

Results

**Figure 1:**
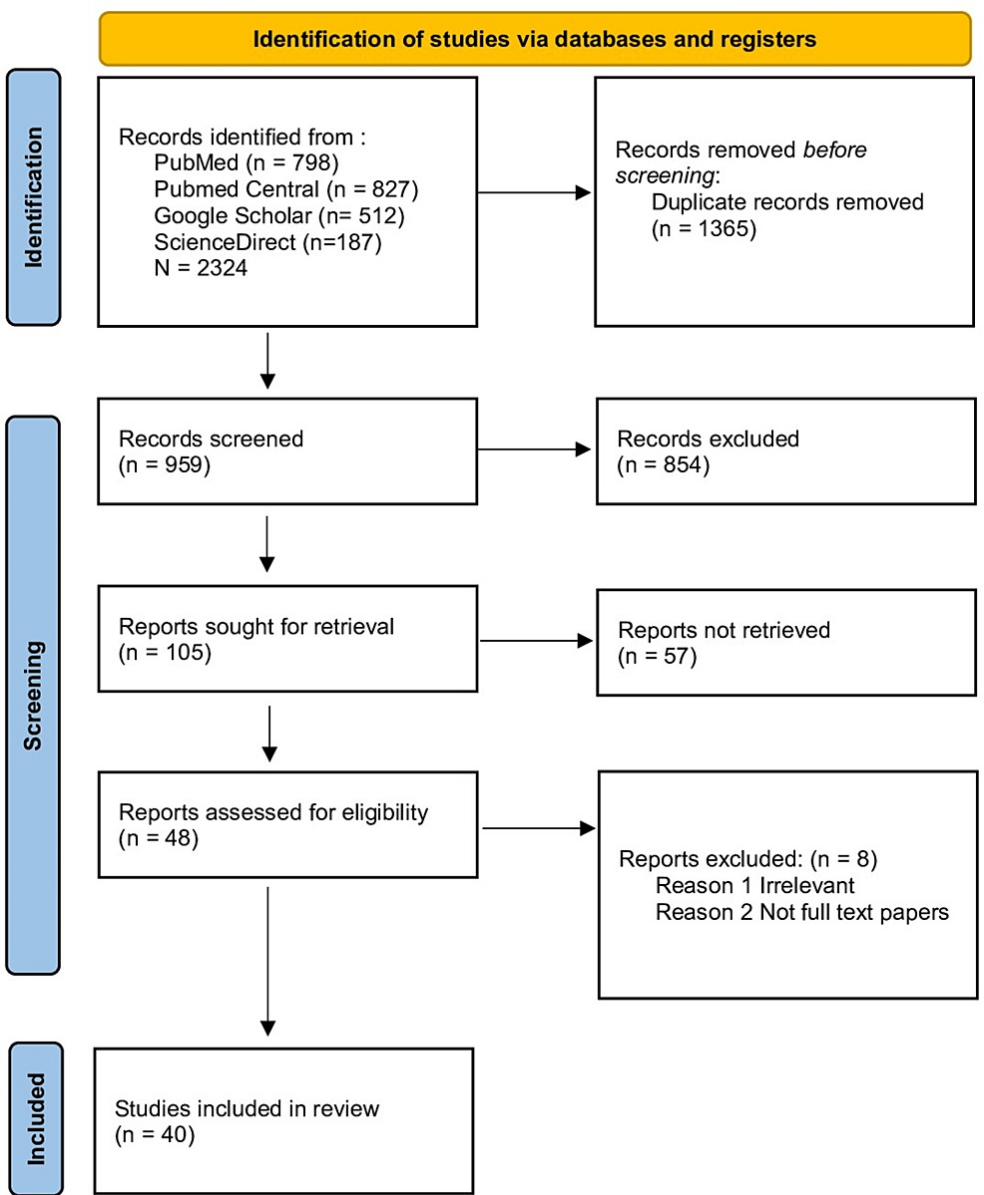
PRISMA 2020 flow diagram for the systematic review PRISMA: Preferred Reporting Items for Systematic Reviews and Meta-Analyses

Initially, 2324 studies were identified in the database search. However, by applying filters based on specific inclusion criteria - including being in the English language, having been published within the past 30 years, involving human subjects, encompassing clinical trials, various types of reviews, and observational studies - the initial number of studies was whittled down to 959. After further scrutiny and quality assessment, reports assessed for eligibility were reduced to 48. Among those, 40 studies presented compelling evidence of a complex relationship between CN and diabetes, as illustrated in (Figure 1).

Pathophysiology of CN

CN is an intricate and perplexing condition characterized by advancing deformity, joint destruction, and neuropathy. Understanding the pathophysiology of CN is vital for active management and prevention, predominantly in diabetic patients most commonly affected by this condition. Some fundamental mechanisms contributing to CN development involve neuropathy, autonomic dysfunction, repetitive microtrauma, hyperglycemia, inflammation and inflammatory states, advanced glycation end products, and microvascular changes. The hallmark feature of diabetes is hyperglycemia and insulin dysfunction [6]. When untreated, it results in macrovascular complications such as atherosclerosis, peripheral artery and venular diseases, cerebrovascular pathologies, and microvascular changes causing diabetic retinopathy, diabetic nephropathy, and diabetic sensory neuropathy. A central component of CN is sensory neuropathy, the primary trigger for the condition [5]. Neuropathy leads to the loss of protective pain sensations, causing individuals not to experience the typical pain response when their joints are subjected to minor injuries or stress. Such sensation loss leads to further damage as the individuals continue to walk and bear weight on an injured joint [7].

Along with sensory loss, impairment of proprioception can also be seen. Due to this, the individual cannot perceive movement and position of one's limb, resulting in difficulty identifying trauma and stress experienced by joints, worsening the problem. Due to this absence of pain and proprioception, individuals with neuropathy may continue to be involved in activities and movement, exposing their joints to microtrauma. Over time, the accumulation of these microtraumas promotes worsening damage, as seen in CN [3]. CN is associated with microvasculature changes, including impaired blood flow regulation and endothelial dysfunction. The endothelium consists of a single layer of cells lining blood vessels, critical in regulating blood flow and vascular tone. Prolonged hyperglycemia, as seen in diabetes, leads to endothelial dysfunction, resulting in impaired constriction responses and vasodilation, impacting healthy blood flow regulation mechanisms [8]. This impact on healthy blood flow regulatory mechanism in the microvasculature affects the delivery of nutrients and oxygen to the affected tissues, predominantly the joints. An adequate oxygen and nutrient supply is vital for bone health and remodeling. However, due to dysregulation of the blood flow mechanism, poor oxygenation and nutrient supply disrupt the balance between bone formation and resorption. Such disruption of balance leads to decreased bone strength and density, contributing to instability and bone deformity, as seen in diabetic patients with CN [9,10].

Diabetes and Pathophysiology

**Figure 2:**
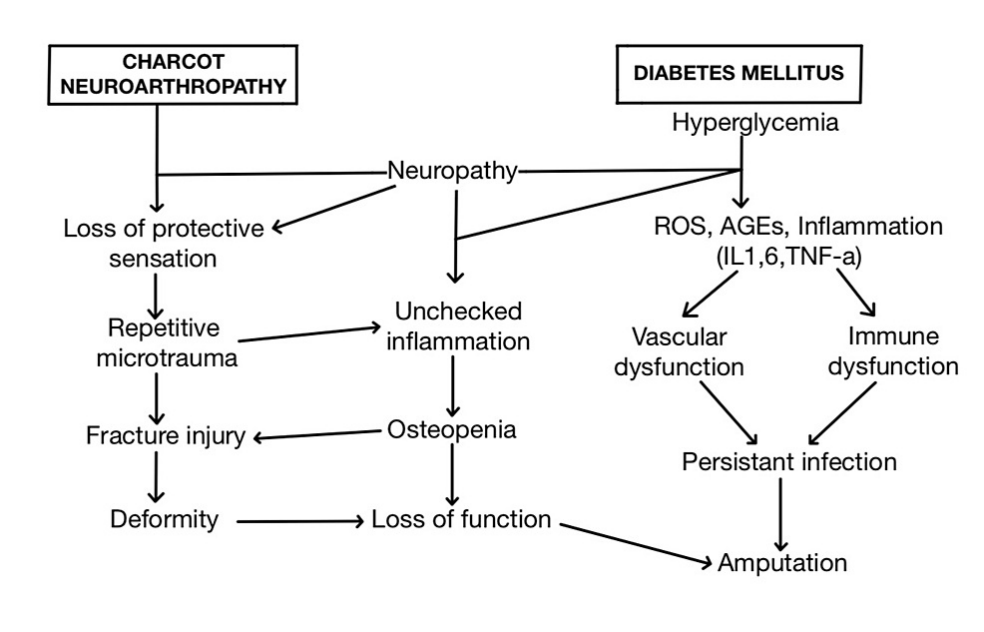
Relationship between diabetes and the pathophysiology of Charcot Neuroarthropathy (CN) Self-created

Diabetes mellitus is a metabolic disorder characterized by chronic hyperglycemia, which leads to a series of physiological changes conducive to the development of CN. A critical factor in the pathophysiology of CN is prolonged hyperglycemia. Advanced glycation end products (AGEs) formed by glycation of proteins and lipids due to high glucose levels accumulate in tissues, disturbing the extracellular matrix proteins and structural integrity of collagen. This glycation process weakens and stiffens tissues, making them more prone to injury, including the joints. Sensory neuropathy is an essential element of CN pathophysiology. This diabetes-induced nerve damage due to chronic hyperglycemia damages the tiny nerve fibers responsible for sensory perception. This loss of protection pain sensation subjects individuals with CN to not experience pain when their joints are under stress, enabling further damage [6]. Chronic high glucose blood sugar levels lead to microvascular changes and complications, such as impaired blood flow regulation and endothelial dysfunction, resulting in poor nutrient supply and oxygenation, contributing to the pathophysiology of CN. Characteristic elevated levels of pro-inflammatory cytokines, such as interleukin-6 (IL-6), tumor necrosis factor-alpha (TNF-α), interleukin-1 beta (IL-1β), and monocyte chemoattractant protein-1 (MCP-1) illustrates a state of chronic low-grade inflammation, impairing immune function and improper execution of lymphocytes, macrophages, and neutrophils [11-13]. These cells are vital for tissue repair and defense against pathogens, leading to increased susceptibility to infections, impaired wound healing, and lowered natural immune response. Due to compromised immunity in diabetic individuals, conditions such as diabetic foot infections are of significant concern in CN [14]. Diabetes causes an imbalance between bone formation and resorption, leading to osteopenia. Due to the amplified bone resorption process, bones are more susceptible to fractures as bone density is decreased. Hormonal imbalances due to diabetes lead to decreased insulin-like growth factor 1 (IGF-1) levels, weakening the bones and making joints more susceptible to damage [15]. At the molecular and cellular level, activation of the receptors for advanced glycation end products (RAGE) and nuclear factor kappa B (NF-κB) signaling advances the development of neurovascular dysfunction, neuropathy, and upregulation of pro-inflammatory cytokines [16]. Figure 2 summarizes the intricate relationship between diabetes and the pathophysiology of CN.

Clinical Features and Diagnosis

The most prominent clinical features in CN are swelling and erythema. Edema in CN is marked and disproportionate to the degree of trauma or injury. This swelling is due to an inflammatory reaction within the surrounding tissues and joints [17]. Skin temperature is also elevated over the affected joint. This increased temperature results from the heightened blood flow to the area and the inflammatory process. Infrared thermography objectively measures this temperature rise, which aids in assessing and diagnosing CN. This clinical feature is essential for distinguishing CN from other musculoskeletal conditions resembling similar clinical manifestations [18]. Sensory neuropathy leads to the loss of protective pain sensations, causing individuals not to experience the typical pain response when their joints are subjected to minor injuries or stress. Such sensation loss leads to further damage as the individuals continue to walk and bear weight on an injured joint. Such neurological manifestation poses a significant challenge in the early diagnosis of CN and delays in clinical presentation evaluation. Therefore, clinicians must rely on imaging findings and other clinical features to diagnose CN without pain. The most common consequence of CN includes advancing joint deformities. These deformities include structural abnormalities within the affected foot, midfoot collapse, and rocker-bottom deformity. Abnormal weight-bearing patterns and gait are the classical symptoms connecting joint deformities and further progression of the condition, increasing the risk of complications [19]. Painless neuropathic joints, a hallmark of CN, are the most confusing feature where pain is absent in individuals with significant joint damage. Painless neuropathic joints have a direct correlation with loss of pain sensation. Due to this loss of sensation, patients do not seek medical attention as they do not experience discomfort or pain, presenting a significant challenge in the early diagnosis of CN. Therefore, CN is often detected at an advanced stage, when deformities have already developed [20]. CN's clinical presentation, which includes erythema, increased skin temperature, and swelling, can mimic the signs of local infection. As the clinical features of infections and CN can be markedly similar, such overlay causes significant diagnostic challenges. Distinguishing between infection and CN is vital, as treatment and management approaches differ significantly in these conditions. An incorrect diagnosis may result in the provision of unsuitable treatments, exacerbating the issue [21]. Development of CN is gradual and insidious in progression, making the condition challenging in terms of diagnosis. This unique feature of gradual onset leads to improper history taking, as patients find it difficult to provide reliable data about when the initial injury or joint damage began. CN's insidious but slow development presents diagnostic challenges because it is often discovered later when deformities are already established. The paradox of painlessness, the gradual onset of the condition, and the need for differentiation from infections focus on the complexities of diagnosing CN in diabetic patients. Distinguishing these features and various diagnostic methods is crucial for the timely diagnosis and management of CN in individuals with diabetes [22].

Various diagnostic methods for confirming CN in diabetic patients involve magnetic resonance imaging (MRI), plain radiographs, infrared thermography, comprehensive clinical examination, laboratory tests, neuropathy assessment, and biopsy. Both soft tissues and bone structures can be visualized in MRI, an advanced imaging modality. MRI provides an all-inclusive view of the affected foot's inflammatory processes and anatomical changes, helping diagnose and manage treatment decisions. Plain radiographs, such as X-rays, play a fundamental role in diagnosing CN. They actively reveal structural changes within the affected foot, such as fractures, bone resorption, and joint dislocations [23]. Infrared thermography is a non-invasive procedure used to measure temperature differences between the two feet. This method is predominantly valuable in identifying the characteristic feature of CN, that is, the increased localized temperature in the affected foot. This method helps calculate the temperature variance, assisting in diagnosis and monitoring. A detailed clinical assessment is crucial for diagnosing CN. It involves meticulously examining the affected foot, focusing on various clinical features such as erythema (redness), temperature changes, and joint deformities. Clinicians cautiously observe these clinical features to determine the presence and severity of CN [24]. Laboratory tests are essential in the diagnostic process to rule out associated infections or other conditions that may mimic CN's clinical presentation. Complete blood counts (CBC) and inflammatory markers are done to recognize potential infections and appraise the patient's overall health [25]. Assessing the severity of neuropathy is important in diagnosing CN as it is robustly linked to sensory neuropathy. Diagnostic tools such as nerve conduction studies and 10-gram monofilament tests are done. Nerve conduction studies estimate nerve function and the presence of neuropathy, while the 10-gram monofilament test appraises the ability to see pressure at specific points on the foot [26]. A bone biopsy may be considered when other diagnostic methods are unsatisfying and uncertain. Biopsy, as an invasive procedure, is considered a last resort, which involves removing a small sample of bone tissue from the affected foot for examination and confirmation of the diagnosis of CN [24].

Epidemiology and Risk Factors

CN is a relatively rare but severe complication of diabetes. Its occurrence differs depending on the geographic region and population studied. In individuals with diabetes, the reported prevalence of CN ranges from 0.08% to 13%. However, this wide range can be due to differences in healthcare accessibility, diagnostic criteria, and the duration of diabetes in the studied population [27]. Several risk factors, such as duration of diabetes, type of diabetes, and glycemic control, contribute to the development of CN in diabetic patients. The incidence of CN is higher in type 1 diabetes due to the longer duration of the disease and earlier onset. Persistent exposure to hyperglycemia and accompanying complications, such as sensory neuropathy, extensively influences CN development. High HbA1c levels indicate poor glycemic control, contributing to vascular changes and neuropathy that underlie CN [28].

Complications and Outcomes

CN in diabetic patients is associated with several potential complications, such as foot ulcers due to loss of protective sensation. These ulcers can become infected and further cause an advanced array of complications. Weakened bones in CN are disposed to dislocations and fractures, leading to impaired mobility. Severe cases of CN can compel partial or complete foot amputations to prevent the spread of infection and preserve the patient's overall health [29].

Impact on Quality of Life and Prognosis

The complications associated with CN, such as infections, foot ulcers, and amputations, can radically reduce independence, mobility, and quality of life. Moreover, the necessity for long-term management and its chronic nature can be psychologically and emotionally burdening for patients. The effectiveness of the treatment and the prognosis largely depend on the stage at which CN is diagnosed. Early diagnosis and intervention can lead to better outcomes, while advanced cases may have a more guarded prognosis [30].

Treatment and Management Strategies

The management of CN in diabetic patients involves an amalgamation of conservative and surgical methods. A vital component of conventional CN management is offloading, which comprises orthopedic shoes, customized casts, and removable braces. This method helps healing by redistributing pressure away from the affected foot [31]. Tailored orthopedic shoes are custom-made to the patient's specific foot shape, lowering the risk of pressure ulcer deformities by providing perfect support and proper relocation of pressure on the affected foot [32]. Another effective offloading technique is total contact casting, which incorporates a plaster cast or fiberglass to the lower extremities. This method assigns uniform weight distribution and prevents disproportionate pressure on vulnerable foot areas [33]. An alternative to these plaster casts is removable braces. They allow periodic removal for care and wound inspection, delivering adaptability in the management of CN [34].

Surgical procedures include realignment, osteotomies, and, in severe cases, amputations. In osteotomies, we make precise cuts in the bone to stabilize and realign the affected joints. These procedures correct deformities by fixing mispositioned bones and improving weight distributions [35]. Another procedure to correct deformities and joint instabilities is realignment procedures. This surgical procedure reinstates the original anatomical alignment and proper joint function, reducing the risk of further damage. Amputation may be necessary in severe cases such as extensive bone destruction or uncontainable infection to prevent the spread of infection and preserve the patient's overall health [36]. The management of CN demands a multidisciplinary approach involving various specialists. Podiatrists are vital in caring for CN patients, offering wound care, foot health, and orthotic management expertise. The role of orthopedic surgeons includes surgical management, such as realignment procedures and amputations. Endocrinologists manage the underlying diabetes and glycemic control, which is critical in preventing further complications and recurrence of CN. Wound care specialists are indispensable in caring for infections, ensuring proper wound healing, and foot ulcers, which are common in CN cases [37].

Preventive Measures

A fundamental basis for preventing CN is maintaining strict glycemic control. Managing blood glucose levels within target ranges and consistent monitoring substantially reduces the risk of developing neuropathy and other diabetes-related complications. Maintaining this control involves healthy practice of prescribed oral medications or insulin intake, dietary modifications, and regular blood glucose monitoring [38]. Regular foot examinations by podiatrists, especially for diabetic patients, are crucial in identifying early signs of CN. These examinations encompass the assessment of pulse, temperature, skin condition, and any abnormalities [25]. Education on proper foot care practices, particularly for diabetic patients, is essential. The importance of wearing appropriate footwear, daily personal foot inspections, and the need for regular podiatric check-ups should be taught. On a basic level, appropriate provision of proper guidance over selecting protective and comfortable footwear is essential to reduce the risk of injury and progression of the condition at early stages. Patients with diabetes should wear well-fitting, cushioned shoes that offer comfort, protection, and support. Shoes with a tight fit, high-heeled shoes, or pointed toes should be avoided. Individuals with pre-existing neuropathy or foot deformities should wear custom orthopedic shoes [39]. For early intervention, regular monitoring for vascular changes and neuropathy is essential. Neuropathy can be evaluated through nerve conduction studies and clinical tests such as the 10-gram monofilament test. Checking peripheral pulses and vascular health helps us monitor vascular changes. These preventive measures can help reduce the risk of developing CN in individuals with diabetes. Proactive foot care, education, and awareness are essential components of CN prevention, and they promote long-term outcomes for those at risk [40]. Table 1 summarises all the studies involved in this review article.

**Table 1:** Summarises all the studies involved in the article

Authors	Year	Findings
Rogers LC, et al. [1]	2011	The article addresses the diabetic Charcot foot syndrome, highlighting the importance of early recognition and offloading as chief treatments to prevent deformities. The role of antiresorptive therapy and bone growth stimulation is also mentioned.
Schon LC, et al. [2]	1998	This study aimed at Charcot neuroarthropathy in the foot and ankle, categorizing specific involvement sites and management methods. Surgical interventions like arthrodesis and fusion and nonoperative management are discussed.
Boulton AJ, et al. [3]	2008	This article tells us the serious modules of an inclusive foot examination for adult diabetic patients. Peripheral neuropathy, deformities, and trauma are the most common primary causes. The inclusion of history, general inspection, dermatological and musculoskeletal assessments, and neurological assessment is also detailed.
Jeffcoate WJ, et al. [4]	2005	Charcot neuroarthropathy is typically portrayed by peripheral neuropathy and abnormal foot biomechanics. Mechanisms involve inflammatory reaction, with increased expression of proinflammatory cytokines ultimately resulting in increased osteoclastogenesis, bone lysis, and further fractures.
Pierre-Jerome C, et al. [5]	2022	This book broadly covers Charcot neuroarthropathy in diabetic patients, comprising pathophysiology, biomechanics, epidemiology, socioeconomic impacts, radiological findings, and differential diagnosis, emphasizing magnetic resonance imaging. The relationship between diabetes-induced peripheral neuropathy and the development of Charcot neuroarthropathy is also covered.
Singh VP, et al. [6]	2014	High glucose levels in diabetes cause the formation of harmful advanced glycation end products through protein glycation, contributing to various diabetic complications, including cataracts, nephropathy, neuropathy, and cardiomyopathy.
Gomatos EL, et al. [7]	2022	This review covers sensory neuropathy, involving its varied causes, prevalence, clinical features, diagnostic methods, treatments, and complications. Evaluation involves neurophysiological tests, skin biopsies, and imaging.
Hadi HA, et al. [8]	2007	Diabetes and insulin resistance lead to a sequence of endothelial dysfunctions, which can result in the development of atherosclerosis. Microalbuminuria is also a risk factor for cardiovascular disease in diabetic and hypertensive patients. Controlling hyperglycemia is the best approach to enhance endothelial function and prevent cardiovascular complications in diabetes.
Brem H, et al. [9]	2007	This study tells us about Diabetic foot ulcers. Mechanisms like decreased blood flow and angiogenesis contribute to poor wound healing. Combined approaches, including off-loading and Food and Drug Administration-approved biological therapies, are essential for treatment.
Mabilleau G, et al. [10]	2008	This study found that patients with acute Charcot's osteoarthropathy had monocytes capable of segregating into highly active osteoclasts when cultured with macrophage colony-stimulating factor. This suggests that increased osteoclastic activity in Charcot's osteoarthropathy is mediated through receptor activators of nuclear factor kappa-B ligand-dependent and receptor activators of nuclear factor kappa-B ligand-independent pathways, possibly involving pro-inflammatory factors. This study focuses on the potential for using receptor activators of nuclear factor kappa-B ligand inhibition in Charcot's foot treatment and suggests that anti-tumor necrosis factor strategies could be beneficial.
Hotamisligil GS, et al. [11]	1993	Increased tumor necrosis factor-alpha expression in obesity is associated with the onset of insulin resistance and reduced glucose uptake and utilization in tissues.
Mandrup-Poulsen T. [12]	2012	This review discusses the role of pro-inflammatory cytokines in type 1 diabetes, focusing on tumor necrosis factor-α and interleukin-1. These cytokines play a role in type 1 diabetes pathogenesis and beta-cell damage. Their blockade in clinical trials has shown only modest efficacy, suggesting the need for better timing and combination therapy. The study also stresses the importance of further research to understand cytokine mechanisms better and develop effective treatments for type 1 diabetes.
Rotter V, et al. [13]	2003	This review discusses the role of pro-inflammatory cytokines, particularly Interleukin-6, tumor necrosis factor-α, and interleukin-8, play a significant role in the induction of insulin resistance in adipose cells.
Donath M, et al. [14]	2011	Studies tell us about the part of inflammation in the pathogenesis of type 2 diabetes, forming its connection to obesity and insulin resistance.
Warren S. Browner [15]	2001	This study found osteoprotegerin serum levels are higher in women with diabetes. These levels are also higher in those who died from cardiovascular disease compared to control subjects. These links were independent of age, body mass index, and other cardiovascular risk factors.
Tóbon-Velasco JC, et al. [16]	2014	The binding of ligands to receptors for advanced glycation end-products causes increased oxidative stress and triggers nuclear factor-kB activation, leading to cell damage. This emphasizes on the critical link between inflammation and oxidative stress in neurodegeneration mediated by receptors for advanced glycation end-products.
Madan SS, et al. [17]	2013	Various investigations for early diagnosis and treatment of Charcot neuroarthropathy in people with diabetes are essential to minimize its complications. Differential diagnosis is essential, especially to distinguish Charcot neuroarthropathy from osteomyelitis, and emerging imaging techniques like hybrid positron emission tomography show promise.
Papanas N, et al. [18]	2013	The Charcot foot is a complication of diabetes mellitus associated with peripheral neuropathy and autonomic neuropathy, leading to increased blood flow and bone resorption. The condition can be classified based on clinical stage (acute and chronic), anatomical involvement, and natural history (development, coalescence, and consolidation).
Lipsky BA, et al. [19]	2020	The International Working Group on the Diabetic Foot published updated guidelines on diagnosing and treating foot infections in diabetic people. The guidelines underline the importance of suitable empirical antibiotic therapy based on the clinical condition and causative pathogens. Surgery may be required for severe infections, and bone resection for osteomyelitis is considered.
Dardari D. [20]	2020	This clinical study of Charcot neuroarthropathy gives us an overview of pathophysiology, clinical presentation, and treatment.
Fierheller M, et al. [21]	2010	This clinical study validates using a handheld infrared thermometer in chronic wound care. In non-infected cases, the temperature difference between peri-wound skin and a control site was <2°F. In infected cases, peri-wound skin temperature was elevated by >2°F. The study underlines the importance of patient-specific factors using clinical judgment alongside infrared thermometry.
Vopat ML, et al. [22]	2018	This study shows that diagnostic challenges often lead to delayed treatment, accenting the need for better screening methods. Early detection and intervention are crucial to prevent foot deformities and severe complications.
Rosskopf AB, et al. [23]	2019	The review is a pictorial review that illustrates the different stages of the disease, its clinical presentation, and typical imaging findings. It highlights the effectiveness of magnetic resonance imaging in assessing complications, such as soft tissue infections and osteomyelitis, and provides visual examples of various imaging features.
Molines L, et al. [24]	2010	Charcot neuro-osteoarthropathy is a severe complication triggered by local inflammation, accelerating foot deformity. This discovery offers new potential treatment strategies, although off-loading remains essential.
Lipsky BA, et al. [25]	2016	Diabetic foot infections should be clinically diagnosed, and suspected osteomyelitis should be confirmed by a probe-to-bone test and elevated inflammatory markers. Obtaining microbiological cultures of tissue specimens from infected wounds is crucial for identifying causative microorganisms. Surgical consultation is recommended in severe cases.
Wang F, et al. [26]	2017	The study inspected the diagnostic correctness of monofilament tests in revealing diabetic peripheral neuropathy. The review determined that the clinical use of monofilament tests for diabetic peripheral neuropathy evaluation cannot be strongly endorsed.
Salini D, et al. [27]	2018	The review found a commonness of Charcot arthropathy in patients with type 2 diabetes and peripheral neuropathy. A population over 50 aided the raised Charcot arthropathy frequency in the study.
Wu Y, et al. [28]	2014	This review explores the raised occurrences of type 2 diabetes due to genetic, environmental, and lifestyle factors. A study of various treatment options, including recognized drugs and evolving therapies like sodium-glucose transport protein 2 inhibitors and stem cell educator therapy, is also discussed.
Wu SC, et al. [29]	2007	This review stresses the expanding commonness of diabetic foot ulcers, highlighting their capacity for infection and lower extremity amputation. The review also discusses advanced wound healing modalities, including growth factors and negative pressure wound therapy.
Wang X, et al. [30]	2022	Diabetic foot ulcers are severe complications of diabetes. They are linked with infections, amputations, and mortality. The complexity of diabetic foot ulcers advance and relapse even after healing, the need for effective preventive measures, and the importance of a suitable classification system to aid clinical diagnosis and management are also discussed.
Armstrong DG, et al. [31]	1998	The study reveals a clear connotation between the depth, infection, and ischemia of diabetic foot wounds and an increased risk of amputation.
Wukich DK, et al. [32]	2008	Current understanding of Charcot arthropathy, from historical perspectives to clinical presentation, anatomical classifications, and treatment strategies, is discussed in this study.
Jeffcoate WJ, et al. [33]	2016	The paper suggestions direction for scheming and conveying clinical studies on the prevention and management of foot ulcers in diabetes. A 21-item checklist to assess the quality of work in the diabetic foot ulcer specialty is included by the authors.
Wrobel JS, et al. [34]	2010	This review discusses gait changes in individuals with diabetes, marked by wider gait, slower walking, and prolonged double support time. The influence of glycosylation on the lower extremities, resulting in various soft tissue changes, is also studied.
Hintermann B, et al. [35]	2004	This study highlights the importance of mimicking ankle anatomy and biomechanics for successful total ankle arthroplasty. The HINTEGRA ankle prosthesis shows promising early results, with some complications mainly occurring in the first year, especially in posttraumatic osteoarthrosis cases.
Berli M, et al. [36]	2017	This study found that treating osteomyelitis within the Charcot region on the foot often leads to more high-level amputations, extended antibiotic treatment, and immobilization. Patients with osteomyelitis outside the Charcot region who underwent amputation had shorter antibiotic therapy. The study recommends treating osteomyelitis outside the Charcot region with primary amputation when necessary.
Ran XW, et al. [37]	2012	Diabetic peripheral artery disease and foot ulcers are significant health concerns for diabetes patients, leading to hospitalizations and lower extremity amputations. Establishing multidisciplinary teams is crucial for enhancing patient survival and outcomes.
Armstrong DG, et al. [38]	2020	This study compared the 5-year mortality rates of diabetic foot complications, including Charcot arthropath, diabetic foot ulcer, and lower extremity amputation, with cancer mortality rates. These findings show the significant burden of diabetic lower extremity complications, suggesting that diabetic foot ulcer and lower extremity amputaare not merely indicators of poor health but are autonomous risk factors for premature death.
Song K, et al. [39]	2023	The review deliberates the position of studying the needs and experiences of patients when scheming medical devices and technologies. It accentuates the significance of user-centered design, involving patients in the improvement process and modifying solutions to individual needs.
Perkins BA, et al. [40]	2010	This study inspected the analytical value of the monofilament examination for diabetic neuropathy occurrence. It discovered that a monofilament score of ≤5 correct responses out of 8 was connected with a high 4-year risk of developing neuropathy, while scores of 6-8 correct responses implied a lower risk.

## Conclusions

CN is a multifaceted and weakening condition that creates a substantial challenge, particularly in the diabetic population. This comprehensive study has shed light on the multifaceted relationship between CN and diabetes, discovering the pathophysiology, clinical presentation, diagnosis, management, complications, and preventive measures linked with this condition. The pathophysiology of CN is multi-layered, surrounding sensory neuropathy, repetitive trauma, hyperglycemia, inflammation, advanced glycation end products (AGEs), and microvascular changes. Diabetes plays a cardinal role in initiating and continuing these pathophysiological processes, ultimately contributing to the development of CN. The clinical features and diagnosis of CN in diabetic patients exhibit distinctive challenges due to the painless nature of the condition. Diagnostic methods, such as clinical assessments and imaging, highlight the value of accurate and timely diagnosis. The review has emphasized the occurrence of CN in diabetic populations, stating various risk factors.

Additionally, it has explained the conceivable complications linked with CN, including infections, deformities, and foot ulcers impacting patients' long-term prognosis. The management of CN encompasses a mixture of conservative approaches and surgical interventions. A multidisciplinary team including endocrinologists, wound care specialists, podiatrists, and orthopedic surgeons is crucial for all-inclusive care due to the intricacy of CN and the comorbidities in diabetic individuals. Various preventive measures, which are crucial for mitigating the risk of CN in diabetic individuals, are also discussed. Intensive care for vascular changes and neuropathy is helpful in early intervention of CN development. Overall, this complete review has provided an organized and detailed understanding of the complex relationship between CN and diabetes.

## References

[1] Rogers LC, Frykberg RG, Armstrong DG (2011). The charcot foot in diabetes. Diabetes Care.

[2] Schon LC, Easley ME, Weinfeld SB (1998). Charcot neuroarthropathy of the foot and ankle. Clin Orthop Relat Res.

[3] Boulton AJ, Armstrong DG, Albert SF (2008). Comprehensive foot examination and risk assessment: a report of the task force of the foot care interest group of the American Diabetes Association, with endorsement by the American Association of Clinical Endocrinologists. Diabetes Care.

[4] Jeffcoate WJ, Game F, Cavanagh PR (2005). The role of proinflammatory cytokines in the cause of neuropathic osteoarthropathy (acute charcot foot) in diabetes. Lancet.

[5] Pierre-Jerome C, Kettner NW (2022). Chapter 8 - Differential diagnosis in charcot neuroarthropathy. The Essentials of Charcot Neuroarthropathy.

[6] Singh VP, Bali A, Singh N, Jaggi AS (2014). Advanced glycation end products and diabetic complications. Korean J Physiol Pharmacol.

[7] Gomatos EL, Rehman A (2023). Sensory Neuropathy. http://www.ncbi.nlm.nih.gov/books/NBK559020/.

[8] Hadi HA, Suwaidi JA (2007). Endothelial dysfunction in diabetes mellitus. Vasc Health Risk Manag.

[9] Brem H, Tomic-Canic M (2007). Cellular and molecular basis of wound healing in diabetes. J Clin Invest.

[10] Mabilleau G, Petrova NL, Edmonds ME, Sabokbar A (2008). Increased osteoclastic activity in acute charcot’s osteoarthopathy: the role of receptor activator of nuclear factor-kappaB ligand. Diabetologia.

[11] Hotamisligil GS, Shargill NS, Spiegelman BM (1993). Adipose expression of tumor necrosis factor-alpha: direct role in obesity-linked insulin resistance. Science.

[12] Mandrup-Poulsen T (2013). Interleukin-1 antagonists and other cytokine blockade strategies for type 1 diabetes. Rev Diabet Stud.

[13] Rotter V, Nagaev I, Smith U (2003). Interleukin-6 (IL-6) induces insulin resistance in 3T3-L1 adipocytes and is, like IL-8 and tumor necrosis factor-alpha, overexpressed in human fat cells from insulin-resistant subjects. J Biol Chem.

[14] Donath MY, Shoelson SE (2011). Type 2 diabetes as an inflammatory disease. Nat Rev Immunol.

[15] Browner WS, Lui LY, Cummings SR (2001). Associations of serum osteoprotegerin levels with diabetes, stroke, bone density, fractures, and mortality in elderly women. J Clin Endocrinol Metab.

[16] Tóbon-Velasco JC, Cuevas E, Torres-Ramos MA (2014). Receptor for AGEs (RAGE) as mediator of NF-kB pathway activation in neuroinflammation and oxidative stress. CNS Neurol Disord Drug Targets.

[17] Madan SS, Pai DR (2013). Charcot neuroarthropathy of the foot and ankle. Orthop Surg.

[18] Papanas N, Maltezos E (2013). Etiology, pathophysiology and classifications of the diabetic charcot foot. Diabet Foot Ankle.

[19] Lipsky BA, Senneville É, Abbas ZG (2020). Guidelines on the diagnosis and treatment of foot infection in persons with diabetes (IWGDF 2019 update). Diabetes Metab Res Rev.

[20] Dardari D (2020). An overview of charcot’s neuroarthropathy. J Clin Transl Endocrinol.

[21] Fierheller M, Sibbald RG (2010). A clinical investigation into the relationship between increased periwound skin temperature and local wound infection in patients with chronic leg ulcers. Adv Skin Wound Care.

[22] Vopat ML, Nentwig MJ, Chong AC, Agan JL, Shields NN, Yang SY (2018). Initial diagnosis and management for acute charcot neuroarthropathy. Kans J Med.

[23] Rosskopf AB, Loupatatzis C, Pfirrmann CW, Böni T, Berli MC (2019). The charcot foot: a pictorial review. Insights Imaging.

[24] Molines L, Darmon P, Raccah D (2010). Charcot's foot: newest findings on its pathophysiology, diagnosis and treatment. Diabetes Metab.

[25] Lipsky BA, Aragón-Sánchez J, Diggle M (2016). IWGDF guidance on the diagnosis and management of foot infections in persons with diabetes. Diabetes Metab Res Rev.

[26] Wang F, Zhang J, Yu J (2017). Diagnostic accuracy of monofilament tests for detecting diabetic peripheral neuropathy: a systematic review and meta-analysis. J Diabetes Res.

[27] Salini D, Harish K, Minnie P (2018). Prevalence of charcot arthropathy in type 2 diabetes patients aged over 50 years with severe peripheral neuropathy: a retrospective study in a tertiary care south Indian hospital. Indian J Endocrinol Metab.

[28] Wu Y, Ding Y, Tanaka Y, Zhang W (2014). Risk factors contributing to type 2 diabetes and recent advances in the treatment and prevention. Int J Med Sci.

[29] Wu SC, Driver VR, Wrobel JS, Armstrong DG (2007). Foot ulcers in the diabetic patient, prevention and treatment. Vasc Health Risk Manag.

[30] Wang X, Yuan CX, Xu B, Yu Z (2022). Diabetic foot ulcers: classification, risk factors and management. World J Diabetes.

[31] Armstrong DG, Lavery LA, Harkless LB (1998). Validation of a diabetic wound classification system. the contribution of depth, infection, and ischemia to risk of amputation. Diabetes Care.

[32] Wukich DK, Sung W (2009). Charcot arthropathy of the foot and ankle: modern concepts and management review. J Diabetes Complications.

[33] Jeffcoate WJ, Bus SA, Game FL, Hinchliffe RJ, Price PE, Schaper NC (2016). Reporting standards of studies and papers on the prevention and management of foot ulcers in diabetes: required details and markers of good quality. Lancet Diabetes Endocrinol.

[34] Wrobel JS, Najafi B (2010). Diabetic foot biomechanics and gait dysfunction. J Diabetes Sci Technol.

[35] Hintermann B, Valderrabano V, Dereymaeker G, Dick W (2004). The HINTEGRA ankle: rationale and short-term results of 122 consecutive ankles. Clin Orthop Relat Res.

[36] Berli M, Vlachopoulos L, Leupi S, Böni T, Baltin C (2017). Treatment of charcot neuroarthropathy and osteomyelitis of the same foot: a retrospective cohort study. BMC Musculoskelet Disord.

[37] Ran XW, Zhao JC (2012). The importance of multidisciplinary foot-care services in the management of diabetic patients with peripheral artery disease and diabetic foot ulcers [Chinese]. Sichuan Da Xue Xue Bao Yi Xue Ban.

[38] Armstrong DG, Swerdlow MA, Armstrong AA, Conte MS, Padula WV, Bus SA (2020). Five year mortality and direct costs of care for people with diabetic foot complications are comparable to cancer. J Foot Ankle Res.

[39] Song K, Chambers AR (2023). Diabetic Foot Care. https://pubmed.ncbi.nlm.nih.gov/31971750/.

[40] Perkins BA, Orszag A, Ngo M, Ng E, New P, Bril V (2010). Prediction of incident diabetic neuropathy using the monofilament examination: a 4-year prospective study. Diabetes Care.

